# Uncovering the persistent gap: The ongoing challenge of integrating sex and gender in biomedical research

**DOI:** 10.7555/JBR.38.20240157

**Published:** 2024-09-18

**Authors:** Janet Delgado, Mónica Cano Abadía, Kaya Akyüz, Melanie Goisauf, David Rodríguez-Arias

**Affiliations:** 1 Department of Nursing, School of Health Sciences, Yamaguchi University Graduate School of Medicine, Ube, Yamaguchi 755-8505, Japan; 2 Department of Philosophy, University of Granada, Campus de la Cartuja s/n, Granada 18071, Spain; 3 ELSI Services & Research Department, Biobanking and BioMolecular Resources Research Infrastructure-European Research Infrastructure Consortium, Graz 8010, Austria

## Introduction

Gender and sex are related to important quality and safety issues in scientific, health, and clinical research. Sex refers to biological characteristics, while gender encompasses the sociocultural norms, identities, and relationships that shape communities and organizations, as well as influence actions, behaviors, contexts, and knowledge. Both gender and sex intersect with other social categories. In this context, in addition to sex or gender, the intersectionality refers to overlapping or interdependent systems of discrimination by more than one factor, such as age, disability, ethnicity, geographic location, socioeconomic status, and sexuality, among others.

Sex bias refers to differential treatment or discrimination based on an individual's biological sex, while gender bias refers to differential treatment or discrimination based on an individual's gender identity or expression. In biomedical research, sex and gender bias may lead to the neglect of issues relevant to specific sexes or genders, or the assumption that findings for one sex or gender are universally applicable. Such biases may lead to inadequate or inappropriate care and solutions.

Women, transgender individuals, and gender-diverse people are often underrepresented in clinical trials^[1–2]^, resulting in a need for more evidence regarding how drugs and treatments affect them. When clinical research includes a diverse population, it ensures that drugs and other interventions are safer and more effective for everyone^[2]^. Sex and gender affect all stages of research, from the strategic concerns of setting goals and developing theories to more mundane tasks of designing questions, creating procedures, and analyzing data. Therefore, we propose the integration of sex- and gender-based and intersectional analysis (SGBIA), which considers the influence of biological sex, sociocultural gender factors, and their intersection with other aspects (*e.g.*, age, ethnicity, or socioeconomic status) in the design, conduct, and interpretation of research studies^[3–4]^. SGBIA works alongside other approaches within a discipline to provide additional "checks and balances" (or filters for bias) in scientific, medical, and engineering research, as well as in policy and practice. Embedding SGBIA into research from the outset may help avoid many problems.

The current perspective aims to highlight the necessity of addressing these issues in a structural and fundamentally practical manner, ensuring meaningful improvements in how these aspects are incorporated into biomedical research.

## Improvements in the incorporation of sex, gender, and intersectionality

Adequate integration of gender, sex, and intersectional issues into biomedical research is critical to improving the quality and safety of clinical research and, consequently, the health of individuals. Specifically, it is crucial to: (1) understand how sex and gender affect disease manifestation and progression, (2) identify differences in health outcomes and treatment responses across diverse genders, (3) develop more personalized and effective treatments for men, women, transgender, intersex, and non-binary individuals, (4) promote health equity by providing tailored healthcare based on sex and gender, and (5) improve the overall quality of biomedical research by accounting for the effects of sex, gender, and intersectional issues on health outcomes.

The more inclusive the population in clinical trials, the greater the safety and efficacy of health technologies, including drugs, vaccines, devices, procedures, and systems. Poor representation of sex-gender data makes it difficult to assess the effects of sex and gender differences on the health and efficacy of interventions across different populations. In clinical trials, potential consequences include skewed or incomplete results, impeding the establishment of evidence-based clinical practice guidelines. Thus, it is crucial to accurately incorporate sex-gender at all stages of the investigation to prevent poor research outcomes and increase their generalizability.

In recent years, there has been a growing understanding of the importance of integrating SGBIA into biomedical research^[5–10]^, accompanied by the development of practical tools to address this need^[11–14]^. While the representation of women, transgender, and gender-diverse individuals has improved^[1–2,15–20]^, the gap persists, and they are still significantly underrepresented in biomedical research.

## Why does the gap persist?

In clinical trial research, it is common to recruit both women and men and to plan for the disaggregated analysis of sex data. However, a common problem in trial design is the lack of strategies to measure and mitigate the risk of sex or gender bias, as well as the lack of consensus on what may be considered a significant sex or gender bias in biomedical research. Researchers do not have tools to assess whether their study is at low, moderate, or high risk of bias. Furthermore, there is no agreement or clear definition of what constitutes a major sex or gender bias in biomedical research. Therefore, the current perspective aims to point out the possible gaps.

While there are some scenarios where gender and sex may not be as important, most scientific research benefits from taking these aspects into account to ensure that results are accurate and relevant to a range of people. ***Table 1*** provides two examples to illustrate when and why gender and/or sex should be included in scientific research, as well as when they are less important.

**Table 1:** Examples of biomedical fields that may or may not require a consideration of sex, gender, and intersectional aspects

Biomedical fields	Consideration
Chronic pain	Consideration of sex, gender, and intersectionality. -Sex: As the neurological and immune systems are influenced by biological factors, including sex hormones, sex is crucial. -Gender: Pain is mostly influenced by gender roles and conventions, cultural variability, *etc*. Gender roles and identity affect how pain is felt, whether or not a patient is willing to disclose it, and how medical professionals treat it. -Race and ethnicity: Two more sociocultural variables that may affect pain management strategies.
Stem cells	Consideration of only sex. By showing potential sex differences in therapeutic capacity as well as in receptor-mediated pathways, sex increases basic knowledge regarding stem cells. In the case of stem cells, it is not necessary to incorporate gender and/or intersectional aspects.
Source: Gendered Innovation in Science, Health & Medicine, Engineering, and Environment^[24]^.	

Most recommendations suggest that sex and gender integration should be considered from the outset, as emphasized by the National Institutes of Health and the European Institute of Women's Health. However, when these aspects have not been integrated from the beginning, what can or should researchers do about it? Once the trial begins, the research project is bound by a strict recruitment protocol approved by a research ethics committee (REC), making it difficult to correct this bias once the clinical trial has started.

This raises the question of why this lack of detailed planning on sex and gender mainstreaming is not carefully detected by RECs. Two main reasons may explain this. On one hand, most study protocols briefly mention the inclusion of the gender dimension in sample calculation and data analysis, which is viewed as "sufficient detail" by a REC. On the other hand, ethics committees do not have specific tools to assess how these dimensions should be integrated (not only sex, but also gender in studies where it is relevant, or intersectional aspects).

## Identifying key aspects to better integrate sex, gender, and intersectional issues in biomedical research

**Figure 1:**
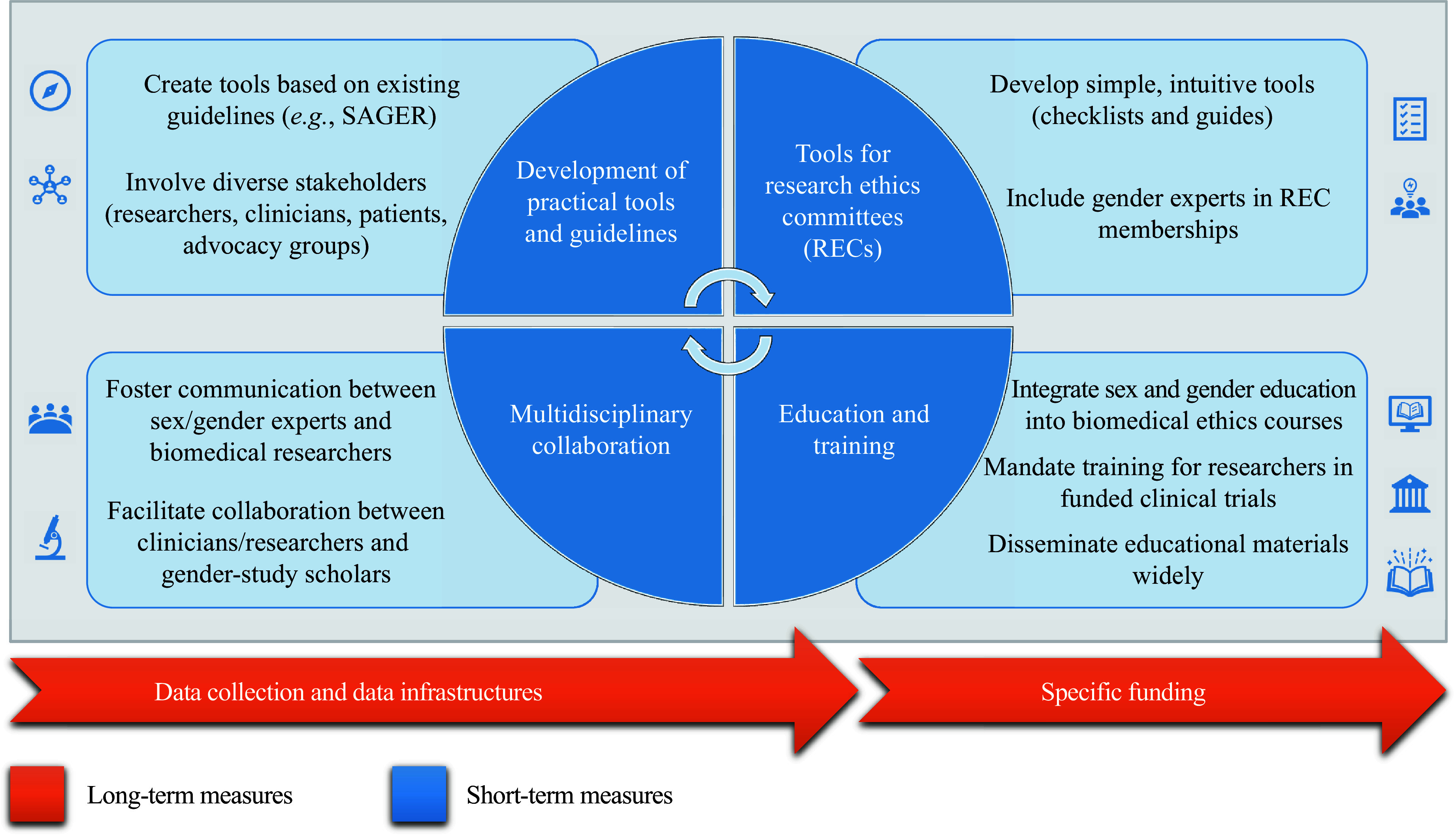
Short- and long-term measures.

It is therefore necessary to identify practical strategies and approaches (not just general guidelines) that facilitate the integration of sex, gender, and intersectional issues in biomedical research. To this end, we summarize the following key actions that may help policymakers address the issues that contribute to perpetuating the sex and gender gap in clinical trials. Here, we describe four recommendations that would be at least partially achievable in the short term (one to two years). However, to be sustainable over time and to achieve a better implementation, it is necessary to promote the creation of infrastructures and specific funds, which are long-term recommendations (***Fig. 1***).

### Short-term measures

**Specific and practical tools to apply the guidelines.** Although there are good guidelines on the incorporation of sex and gender in clinical research, such as the Sex and Gender Equity in Research (SAGER) guidelines^[21]^, the issue of avoiding sex and gender bias is still not satisfactorily addressed in major guidelines, such as the International Ethical Guidelines for Health-Related Research Involving Humans^[22]^, which lacks a specific focus on sex and gender other than guidelines 18 and 19 on women and pregnant women. The need for guidelines has recently been recognized, and efforts are underway^[11–14]^. However, practical tools need to be developed to bridge the gap between guidelines and implementation. To improve the quality and inclusivity of clinical research and ultimately health outcomes for all populations, such guidance should be developed with input from a diverse group of stakeholders, including researchers, clinicians, patients, and advocacy groups.

**Development of tools for RECs.** To promote a better assessment of these aspects by RECs, the development of simple, easy, and intuitive tools, such as checklists and how-to guides, is needed. To our knowledge, no specific tools exist for RECs to assess potential sex and gender bias. In addition, including gender experts as part of the REC membership would also be advisable^[23]^.

**Training of researchers and students in biomedicine.** Basic education on sex and gender issues may be included in biomedical research ethics courses, and mandatory training on these issues should be required before participation in a funded clinical trial. To facilitate a paradigm shift, all undergraduate and postgraduate students must receive education on sex and gender issues in biomedical research^[23]^. To support this effort, it is necessary to develop and widely disseminate practical materials, such as case studies, examples, and easily accessible resources. Many such resources have already been developed by the project Gendered Innovations^[24]^.

**Multidisciplinary collaboration between clinical and gender researchers.** Enhanced and continuous communication of sex and gender experts with biomedical researchers must be fostered^[25]^. Improved multidisciplinary collaboration and opportunities for conversation among clinicians, researchers, and gender scholars would facilitate knowledge transfer and operationalize equity initiatives.

### Long-term measures

**Data collection and data infrastructures.** The lack of available data on sex and gender in healthcare is a significant issue. While there should be institutional support to secure sufficient funding for measures to reduce gender inequalities, it is also crucial to involve healthcare stakeholders to ensure accurate reporting. Transnational guidelines mandating the reporting of sex and gender, *e.g.*, through the realization of the European Health Data Space, may contribute to accurate healthcare data collection.

**Specific funding.** Finally, there is a need for more funding and resources to support research and training programs with an emphasis on sex and gender dimensions. Ensuring the feasibility of such initiatives may help equip researchers and regulators with the knowledge and skills needed to design, conduct, and report clinical trials that are inclusive and representative of diverse populations. By investing in feasible research and training programs, we may improve the quality and inclusivity of clinical research and ultimately improve health outcomes for all populations.

## Challenges and limitations

The proposed actions are not without challenges, and we recognize that difficulties may emerge in developing specific tools to facilitate the appropriate integration of sex and gender dimensions in biomedical research, as well as in integrating practical tools into REC evaluation processes. This is partly due to the lack of standardization that often exists across countries. However, RECs play crucial roles in ensuring that research involving human participants is conducted ethically and responsibly. RECs often provide guidance and education to researchers on ethical principles and best practices in research involving human participants. They may offer workshops, guidelines, or consultations to help researchers navigate ethical issues, including concerns about sex and gender bias. We believe that this is one of the most sustainable ways to ensure change, although we recognize that training researchers will be complex.

## Conclusions

While there have been significant advances in the past decade in incorporating sex, gender, and intersectional aspects into biomedical research, there are still many unaddressed problems that perpetuate the persistent gap. Paying attention to what happens in practice, especially the challenges that both researchers and REC members must face, we have proposed a roadmap for policymakers to consider addressing each of these key aspects. Further research is needed to provide solid evidence and data that support these efforts.

## Fundings

This work was funded by the European Union's Horizon 2020 Research and Innovation Programme under grant No. 896932 (TTV guide TX project) and grant No. 824087 (EOSC-Life).
